# Supplementary material for: The Plasmodium liver-specific protein 2 (LISP2) is an early marker of liver stage development
Source: eLife. 2019 May 16;8:e43362. doi: 10.7554/eLife.43362 (PMC6542585; doi:10.7554/eLife.43362)
Supplement: Supplementary file 1. — The data listed in the table derived from Voorberg et al, 2017. (Sz is schizont; three replicates and Hz is hypnozoite; four replicates) [file elife-43362-supp1.docx]

| **Gene Id** | **Description** | **Sz_1** | **Sz_2** | **Sz_3** | **Hz_1** | **Hz_2** | **Hz_3** | **Hz_4** |
| --- | --- | --- | --- | --- | --- | --- | --- | --- |
| PcyM_MT02100 | unspecified product | 555.5 | 1149.26 | 2189.26 | 12.74 | 45.62 | 9.31 | 20.15 |
| PcyM_0801500 | conserved Plasmodium protein, unknown function | 789.91 | 1691.79 | 446.42 | 15.87 | 8.76 | 32.03 | 16.24 |
| PcyM_0307500 | LISP2; 6-cysteine protein | 608.83 | 806.56 | 1198.55 | 13.02 | 24.71 | 5.16 | 5.13 |
| PcyM_1102600 | TIM9; mitochondrial import inner membrane translocase subunit TIM9, putative | 552.44 | 399.59 | 1615.72 | 35.58 | 0.27 | 1.81 | 8.53 |
| PcyM_1149100 | conserved Plasmodium protein, unknown function | 668.08 | 680.34 | 701.59 | 0.61 | 26.36 | 1.25 | 15.52 |
| PcyM_1433600 | conserved Plasmodium protein, unknown function | 350.8 | 616.47 | 748.46 | 2.23 | 20.1 | 1.5 | 17.54 |
| PcyM_1339400 | hypothetical protein | 617.2 | 476.16 | 905.65 | 16.78 | 5.78 | 6.76 | 11.33 |

**Supplementary file 1**
